# Radical Cure of Corns

**Published:** 1850-07

**Authors:** 


*Radical Cure of Corns.*—In the number of L'Abeille Medicale of the 15th April, M. B. Matton, proposes a mode of curing corns, without a resort to cutting instruments. He advises that the feet be soaked in water for a short time, and the most projecting part of the corn be taken off with a penknife, or with the fingers; a stick of Nitrate of Silver moistened at the free extremity is then to be pressed slightly over the whole surface of hardened cuticle, and even a little beyond on the sound skin. The part to which the caustic is applied, should then be well dried, and let alone for ten days. A very slight and hardly perceptible vestication takes place, which, however, is soon absorbed. At the end of eight or ten days, by making some slight tractions with the fingers, or a pair of dissecting forceps, from the circumference to the centre of the eschar, we may remove, without the slightest pain, the hardened epidermis, so completely as to leave no trace behind. M. Matton pledges himself that those who try his plan will be certainly and radically cured.—*Southern Med. Jour.*

